# The power of light and sound: optoacoustic skin imaging for diabetes progression monitoring

**DOI:** 10.1038/s41377-023-01322-z

**Published:** 2023-11-23

**Authors:** Amanda P. Siegel, Kamran Avanaki

**Affiliations:** 1 Richard and Loan Hill Department of Biomedical Engineering, University of Illinois at Chicago, Chicago, IL USA; 2 Section of Neonatology, Department of Pediatrics, UI Health Children’s Hospital of the University of Illinois at Chicago, Chicago, IL USA; 3 Department of Dermatology, University of Illinois at Chicago, Chicago, IL USA

**Keywords:** Imaging and sensing, Spectrophotometry

## Abstract

Diabetes progression is marked by damage to vascular and neural networks. Raster-scan optoacoustic mesoscopy holds the potential to measure extent of diabetes progression by analyzing changes in skin vasculature.

It is estimated that over half a billion adults worldwide are living with diabetes^1^ including over five million people in the UK^2^ and over 37 million in the US^3^. The costs spent on treating diabetes and its complications is staggering: over $750 billion worldwide and nearly $200 billion in the US alone^4^. Half to two thirds of these costs are attributable to complications of diabetes^5^. Diabetes disrupts angiogenesis which causes damage to large and small blood vessels, leading to a host of complications that include impaired wound healing, cardiovascular complications, and dysregulation of neovascularization causing retinopathy, neuropathy, nephropathy, diabetic microangiopathy, and peripheral vascular disease^6,7^ (Fig. 1).

**Fig. 1:**
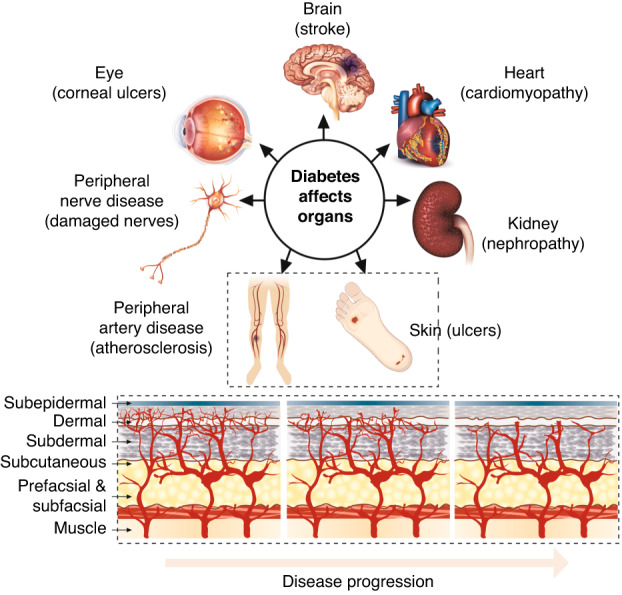
Complications of diabetes stem (top) stem from dysregulation of angiogenesis leading to microangiopathy (bottom). Extent of microangiopathy correlates to relative burden of diabetic complications and disease progression

Because of the fine network of microvasculature that runs through the dermis, researchers have long suspected that the extent of vascular deficiencies associated with diabetes might also be detected or monitored through skin imaging^8^. A range of imaging technologies have been employed for this purpose, including purely optical techniques such as confocal or two-photon microscopy^9,10^, hyperspectral imaging^11,12^, laser speckle contrast imaging^13^, optical coherence tomography^14,15^, nailfold capillaroscopy^16,17^, and a purely acoustic technique, high frequency ultrasound^18–21^. These studies have generally shown they are capable of differentiationg between healthy subjects and persons with diabetes, but have not had sufficient resolution, contrast, or penetration depth to confidently differentiate among people with diabetes exhibiting different complications.

Ultra-wideband raster scan optoacoustic mesoscopy (UWM-RSOM) is particularly valuable for capturing deep microvasculature because it combines optical contrast and ultrasound’s high spatial resolution and penetration depth. Hemoglobin is a highly absorbing chromophore capable of generating a strong optoacoustic signal, enabling RSOM to visualize dermal microvasculature features up to 1.5 mm deep. This suggests that RSOM could potentially image microangiopathy in the skin to not only detect diabetes, but grade the extent of change of microvasculature, which may enable it to grade the expected extent of complications currently or anticipated to be found in a person with diabetes. This is crucially important because current methods for quantifying diabetes complications are very imprecise, relying more on predictions by risk factors and/or assessment of clinical symptoms and signs, such as presence and quality of symptoms.

In a newly published paper in *Light: Science & Applications*, a team led by Vasilis Ntziachristos have demonstrated that RSOM can not only detect but also quantify levels of changes in dermal microvasculature that correlate with extent of complications associated with diabetes. 98 subjects with diabetes and 48 healthy matched controls (HC) were recruited for their study^22^. Approximately half of the subjects with diabetes had no existing complications (NC), one quarter had neuropathy, but no atherosclerotic cardiovascular disease (ASCVD) or peripheral arterial disease (PAD), and one quarter had both neuropathy and ASCVD/PAD. For those with neuropathy but no ASCVD/PAD, the subjects were further divided into those with a low neuropathy score (LN) and those with a high neuropathy score (HN). The authors then went on to demonstrate that the number of small vessels (SVN) in pretibial dermal skin were statistically significantly different between HC and NC (*p* < 0.01), and between NC and LN (*p* < 0.05) and between LN and HN (<0.001). Total blood volume within the dermal layer was similarly statistically significantly different. SVN and total blood volume was also compared between participants with neuropathy with no ASCVD/PAD (NnA) and subjects with neuropathy and ASCVD or PAD (NA). Again, SVN and total blood volume were statistically significantly different (*p* < 0.001). Researchers also analyzed other potential features for their ability to differentiate subjects by extent of disease, looking at number of large vessels and total vessels in the dermal layer, epidermal thickness, and epidermal signal density. Some of these also showed statistical intergroup differences. Mice with and without diabetes were also imaged and a number of the findings were confirmed through histological analysis.

Currently, clinicians collect characteristics which correlate, in a general way, with increases in complications and might predict actual worsening of disease. These characteristics include age, disease duration, body mass index (BMI), glycated hemoglobin (HbA1c), type of diabetes (type 1 or type 2), and sex. Even taken together, and even using advanced AI modeling techniques, these characteristics have not been able to predict onset or likelihood of complications with much success^23^. Thus, not surprisingly, these characteristics did not correlate with the RSOM biomarkers: there was no significant correlation between age, disease duration, HbA1C, and BMI and either SVN or total blood volume^22^.

Looking forward, the method as applied in the study is not yet ready for clinical adoption. Patients were asked to consume no caffeine or food for 4 h before the RSOM measurements and left to relax in a dark room for 5 min prior to imaging, and the room temperature was carefully maintained. Each image took 70 s to acquire, and motion, including arterial pulsation and hearbeat, can lead to inconsistent results, leading to exclusion of RSOM datasets from 8 participants due to serious motion and low image quality. The imaging system was a custom-built in-house portable RSOM imaging system (central frequency 50 MHz) which is not commercially available. Image post-processing was not fully automated. Clearly, modifications to the collection method, instrument, and post-processing analysis would be necessary before clinical implementation. Yet the method holds great promise. Physicians believe that rate of disease progression in a person with diabetes can in many cases be altered by lifestyle changes. A tool for measuring extent of microangiography may offer caregivers a metric to better communicate potential risks to patients with diabetes and help patients implement lifestyle changes before complications worsen. Alternatively, this tool might be able to elucidate other biological risks outside of a person’s control that accelerate complications and disease progression. Diabetes is one of the most significant causes of morbidity worldwide (and, indirectly, mortality). This promising result suggests a possible path forward for utilizing RSOM imaging to enable clinicians, working with persons with diabetes, to reduce the burden of this disease.
